# Metabolic profiling and novel plasma biomarkers for predicting survival in epithelial ovarian cancer

**DOI:** 10.18632/oncotarget.16739

**Published:** 2017-03-31

**Authors:** Hongyu Xie, Yan Hou, Jinlong Cheng, Margarita S. Openkova, Bairong Xia, Wenjie Wang, Ang Li, Kai Yang, Junnan Li, Huan Xu, Chunyan Yang, Libing Ma, Zhenzi Li, Xin Fan, Kang Li, Ge Lou

**Affiliations:** ^1^ Department of Epidemiology and Biostatistics, School of Public Health, Harbin Medical University, Harbin 150086, China; ^2^ Department of Gynecology Oncology, the Tumor Hospital, Harbin Medical University, Harbin 150086, China; ^3^ Harbin Medical University, Harbin 150086, China; ^4^ School of Basic Medical Sciences, Heilongjiang University of Chinese Medicine, Harbin 150040, China

**Keywords:** epithelial ovarian cancer, metabolomics, plasma, survival, prediction

## Abstract

Epithelial ovarian cancer (EOC) is one of the most lethal gynecological malignancies around the world, and patients with ovarian cancer always have an extremely poor chance of survival. Therefore, it is meaningful to develop a highly efficient model that can predict the overall survival for EOC. In order to investigate whether metabolites could be used to predict the survival of EOC, we performed a metabolic analysis of 98 plasma samples with follow-up information, based on the ultra-performance liquid chromatography mass spectrometry (UPLC/MS) systems in both positive (ESI+) and negative (ESI-) modes. Four metabolites: Kynurenine, Acetylcarnitine, PC (42:11), and LPE(22:0/0:0) were selected as potential predictive biomarkers. The AUC value of metabolite-based risk score, together with pathological stages in predicting three-year survival rate was 0.80. The discrimination performance of these four biomarkers between short-term mortality and long-term survival was excellent, with an AUC value of 0.82. In conclusion, our plasma metabolomics study presented the dysregulated metabolism related to the survival of EOC, and plasma metabolites could be utilized to predict the overall survival and discriminate the short-term mortality and long-term survival for EOC patients. These results could provide supplementary information for further study about EOC survival mechanism and guiding the appropriate clinical treatment.

## INTRODUCTION

Epithelial ovarian cancer (EOC) is one of the most life-threatening gynecological malignancies worldwide. Upon diagnosis, patients with ovarian cancer are initially treated with a combination of surgical resection and chemotherapy [1]. Despite initial aggressive treatment, patients are always associated with an extremely poor overall survival (OS); and the average 5-year OS rate for all stages is 45.6% [2]. Currently, several researches have utilized clinical information to predict the prognosis of EOC [3–5]. For example, Barlin et al. developed a nomogram to predict the 5-year disease-specific mortality based on residual disease, stage, tumor histology, age, albumin level, family history of hereditary breast and ovarian cancer syndrome, and physical status [3]. However, due to the heterogeneity of ovarian cancer, it is difficult to predict the clinical outcomes of EOC with simple demographic and clinical characteristics. Therefore, in recent years, with the development of microarray and protein arrays technologies, several studies have been performed to identify the biomarkers from genomics and proteomics to establish prognostic models for ovarian cancer. Yoshihara et al. identified a 126-gene expression signature for predicting OS in high-grade serous ovarian cancer based on primary tumor tissues [6], and Konstantinopoulos et al. performed the gene expression profile of BRCAness and concluded that BRCAness profile was associated with survival in EOC patients [7]. Yang et al. utilized proteins as markers to predict survival based on the reverse-phase protein arrays platform [8]. Although, these studies focus on predicting the survival or progression in EOC patients or high-risk serous ovarian cancer patients, almost all of these studies have been performed on tissues, which were not ideal for survival prediction in clinical practice. Besides, these studies are based on genomics and proteomics techniques. As we know, most genomics and proteomics methods have certain disadvantages, such as low detection efficiency, complex sample preparation procedures, and high cost [9–11]. Therefore, a rapid, high efficiency and economical method for predicting survival of EOC is quite urgent.

Metabolomics is dedicated to the global semi-qualitative assessment of endogenous small molecule metabolites within cells, tissues, and bio-fluids, which is sensitive to reflect the degree of disease progression [12–15]. Compared with genomics or proteomics, metabolomics reflects changes in phenotype and function, which is complementary as “upstream” changes in genes and “downstream” changes in proteins [16]. Metabolomics aims to identify metabolites which are screened and monitored in the urine, feces and tissue samples [17–19]. In light of escalating health care costs and risky invasive procedures, metabolomics can be chosen as a safe yet precise method for monitoring disease prognosis [20, 21]. Some studies demonstrate that dysregulated metabolism is associated with the survival of cancers in humans, highlighting the potential of metabolomic analyses of biological samples for the prognosis of cancers including pancreatic adenocarcinoma, bladder cancer, and colorectal cancer [20, 22, 23]. Besides, it may provide therapeutic avenues to improve patient outcomes. Unfortunately, only Hilvo et al. performed a metabolomics profiling analysis on 158 serum and 112 tissue samples for EOC patients and found that deregulation of 4-Hydroxyphenyllactic acid and 3-Hydroxyisovaleric acid are associated with OS in serum metabolomics study, while high concentration of 3,4-dihydroxybutyric, 2,4-dihydroxybutyric, and adipic acids in tissue are associated with poor OS in tissue [24]. However, this study has not evaluated the predictive performance of these metabolites for EOC survival.

In this study, we mainly aim to explore whether plasma metabolomics profiling could be used for predicting EOC survival and further identifying the potential predictive biomarker/s. Besides, we would like to build up a risk score with demographic and clinicopathological predictors, plus potential biomarkers, and further validate the predictive performance for EOC three-year survival. Finally, we also describe the temporal patterns in relative intensity for the potential predictive biomarkers and further distinguish short-term mortality from long-term survival.

## RESULTS

### Demographic and clinical characteristics of EOC patients

**Figure 1:**
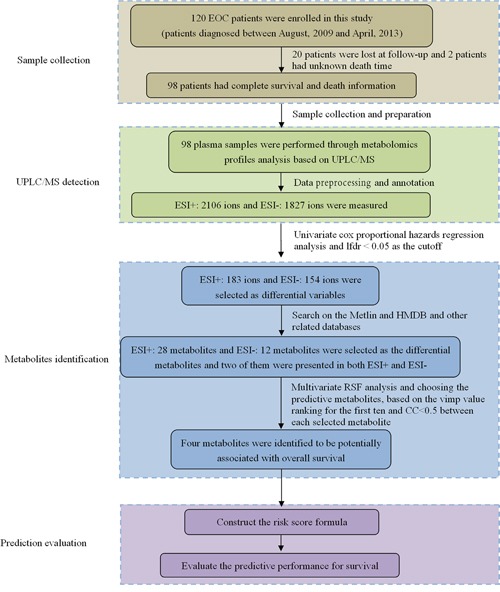
The workflow of this study

Median follow-up time of 98 EOC patients was 37.5 months (range, 1-79 months) in this study. Fifty-four EOC patients died and were with the death time, and forty-four EOC patients were still alive by the end of the last follow-up. The patient-and tumor-related characteristics were listed in Supplementary Table 1. Among the patients, forty-six EOC patients were dead in 3 years after diagnosis, and fifty-two patients still survived after 3 years. The 3-year OS rate was 46.94% in this current study. The specific workflow was displayed in Figure 1.

### EOC survival related metabolites

**Figure 2:**
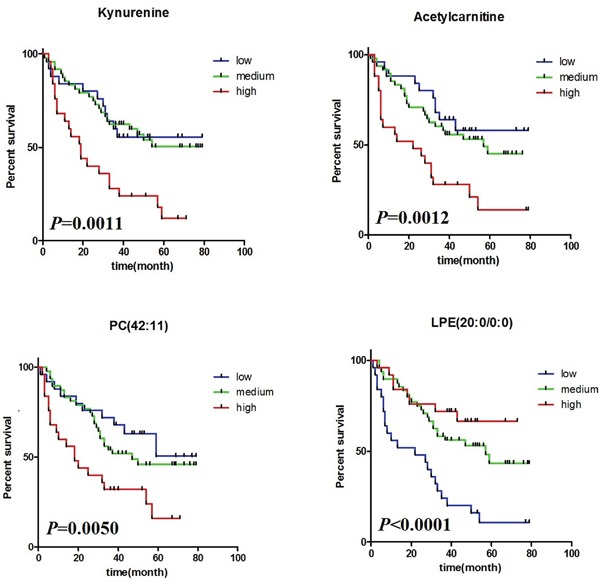
Kaplan-Meier curve and log-rank test comparing the relative intensity of four potential predictive metabolites

Using UPLC/MS system, we generated metabolomic profiles for 98 EOC patients and obtained 2106 ions derived from ESI+ and 1827 ions from ESI-, respectively. The PCA plot revealed that the QC samples were tightly clustered in PCA score plots (Supplementary Figure 1), indicating the robustness of our metabolic profiling platform. Univariate Cox proportional hazards regression analysis was used to identify the association between each ion and OS with the local false discovery rate (lfdr) < 0.05. The selected ions then underwent metabolite identification and were similar to our previous studies [25]. In total, we identified 28 metabolites, related to the OS for EOC in ESI+ mode and 12 metabolites in ESI- mode, and listed these biomarkers in Supplementary Table 2. In addition, Multiple Random Survival Forest (RSF) analysis was used to rank the relative variable importance of survival related metabolites, and top ten metabolites were chosen for further study. We selected four metabolites as the potential predictive biomarkers for survival, based on CC<0.5 between each selected metabolite, which were Kynurenine, Acetylcarnitine, PC (42:11), and LPE(22:0/0:0) (Table 1). In order to clearly visualize the relationship between different scaled relative intensity of each metabolite and survival time, we divided them into low, medium, and high relative intensity groups, based on their corresponding 25^th^ and the 75^th^ percentiles of scaled relative intensity as cutoffs. Kaplan-Meier survival curves and log-rank tests were performed, and the *P* values were 0.0011, 0.0012, 0.0050, <0.0001 for Kynurenine, Acetylcarnitine, PC(42:11), LPE(22:0/0:0), respectively (Figure 2) and suggested poor survival with the increase of Kynurenine, Acetylcarnitine and PC(42:11) and with the decrease of LPE(22:0/0:0).

**Table 1:** Scaled relative intensity of four predictive metabolites significantly associated with overall survival

Metabolite	m/z	RT(min)	Vimp	Coefficient	*P* value	HR	95%CI
Kynurenine	209.0916	3.66	0.012115	0.820	0.0440	3.580	1.833-6.992
Acetylcarnitine	204.1221	1.88	0.010745	0.798	0.0069	3.596	2.195-5.891
PC(42:11)	852.553	13.33	0.00991	0.560	0.0008	1.501	1.154-1.954
LPE(22:0/0:0)	538.3865	12.81	0.00705	-1.185	0.0050	0.262	0.134-0.510

Abbreviations: Measured mass to charge ratio (m/z); Retention time (min, RT); Hazard ratio (HR), Confidence interval (CI); Relative variable importance (Vimp).

### Risk score and establishment

A risk score, defined as a linear combination of the four predictive metabolites, was used to dichotomize the patients into low-risk and high-risk groups using the median risk score as the cut-off. It was established by cox regression coefficients with the scaled relative intensity of these four predictive metabolites (Table 1). The risk scores were as follows:

Risk score=(0.820×Kynurenine)+(0.798×Acetylcarnitine)+(0.560×PC(42:11))-(1.185×LPE(22:0/0:0)). Each metabolite was calculated by their scaled relative intensity.

**Figure 3:**
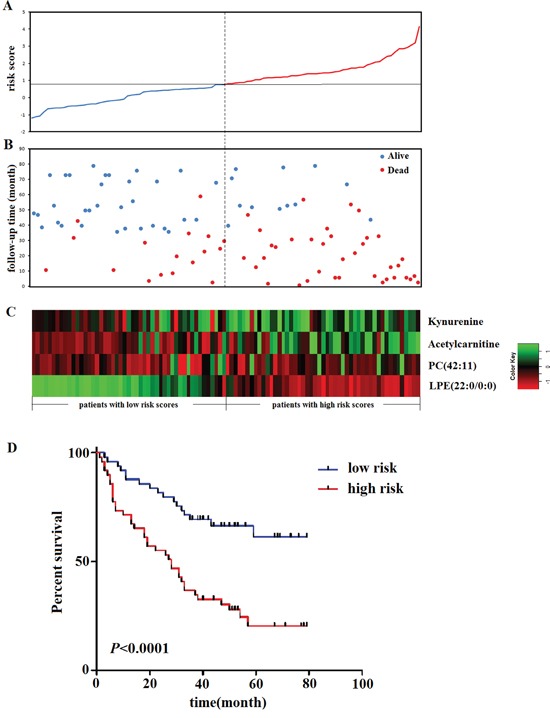
Metabolite-based risk score analysis of EOC patients **(A)** Distribution of the metabolite-based risk scores; **(B)** follow-up time and the status of EOC patients; **(C)** heatmap of predictive metabolites. Rows represent each predictive metabolite and columns represent patients. The dotted line divided patients into low-risk and high-risk groups based on the median risk sore. **(D)** Kaplan-Meier estimates of the survival of the metabolite-based risk score.

According to the risk score and the threshold criteria, all the patients were divided into low-risk (n=49) and high-risk (n=49) groups. Figure 3A showed the distribution of patient risk scores ranking from the lowest risk score to the highest risk score, and the discrimination potential of these four metabolites for the EOC survival, based on the risk scores, was presented in Figure 3B. 32/49 (65.31%) patients who died in three years were correctly classified as low risk patients, and 37/49 (75.51%) alive patients were correctly classified as high risk patients. Heatmap plot of the scaled relative intensity of these four predictors clearly demonstrated that each metabolite could discriminate patients with low risk scores from those with high risk scores (Figure 3C). The statistical difference exists between the low and high-risk subgroups in the OS (*P*<0.0001) (Figure 3D).

### Evaluation of predictive performance of three-year survival

**Figure 4:**
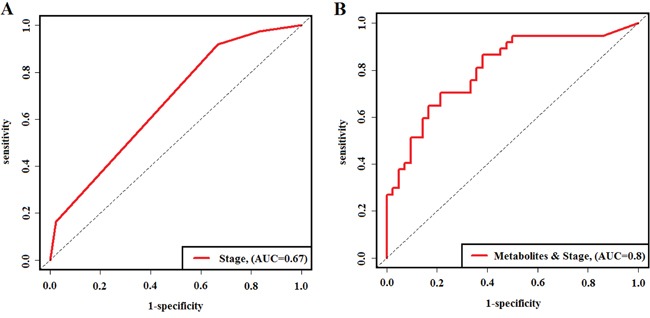
Time-dependent ROC curves evaluating predictive accuracy of three-year survival **(A)** time-dependent ROC curve for pathological stage in the predictive of three-year survival of EOC patients. **(B)** time-dependent ROC curve for risk scores combined the predictive metabolites and pathological stage in the prediction of three-year survival of EOC patients.

Demographic and clinical information were always used to predict the survival in EOC patients, and we explored whether our metabolite-based risk score, together with those factors, could improve the prediction performance. Univariate Cox hazard analysis showed that metabolite-based risk score (HR: 2.661, 95%CI: 1.955-3.623, *P*=8.2×10^−11^), pathological stage (HR: 3.185, 95%CI: 1.774-5.721, *P*=1.1×10^−5^), and cycles of chemotherapy (HR: 0.416, 95%CI: 0.186-0.930, *P*=3.2×10^−2^) presented the statistically significant association with OS. A multivariate analysis on risk score, pathological stage, and cycles of chemotherapy were further conducted. Both risk score and pathological stage still remained statistically associated with OS (Table 2). After that, in order to explore how much predictive performance would be increased with these four metabolites together with pathological stage in comparison to the pathological stage alone, we constructed risk scores that consisted of four metabolites and pathological stage. Time-dependent ROC analysis was used to evaluate the predictive accuracy of three-year survival with pathological stage alone and risk scores (Figure 4). From this result, we could see that the AUC of pathological stage alone and risk scores were 0.67 and 0.80, respectively. The sensitivity and specificity of risk scores were equal to 0.70 and 0.79 based on Youden index [26]. These results indicated that the utility of combination of our biomarkers and clinical factors improved prediction accuracy.

**Table 2:** Univariate and multivariate Cox regression analysis of risk score and clinical factors associated with overall survival

Factors	*P* value	HR	95% CI
Univariate analysis			
Risk score	8.2×10^−11^	2.661	1.955-3.623
Age (<50 vs. ≥50 y)	0.48	1.224	0.689-2.176
Menopause (pre vs. post)	0.14	0.657	0.372-1.161
CA125 (≤500 vs. >500)	0.57	0.857	0.502-1.465
Stage(I vs. II vs. III vs. IV)	1.1×10^−5^	3.185	1.774-5.721
Cycles of chemotherapy (<6 vs. ≥6)	3.2×10^−2^	0.416	0.186-0.930
Multivariate analysis			
Risk score	4.2×10^−4^	3.504	1.746-7.029
Stage(I vs. II vs. III vs. IV)	2.0×10^−3^	9.622	2.292-40.390
Cycles of chemotherapy (<6 vs.≥6)	0.7	0.830	0.325-2.123

Abbreviations: versus (vs); Hazard ratio (HR); Confidence interval (CI).

### Temporal patterns of predictive metabolites in the EOC progression

**Figure 5:**
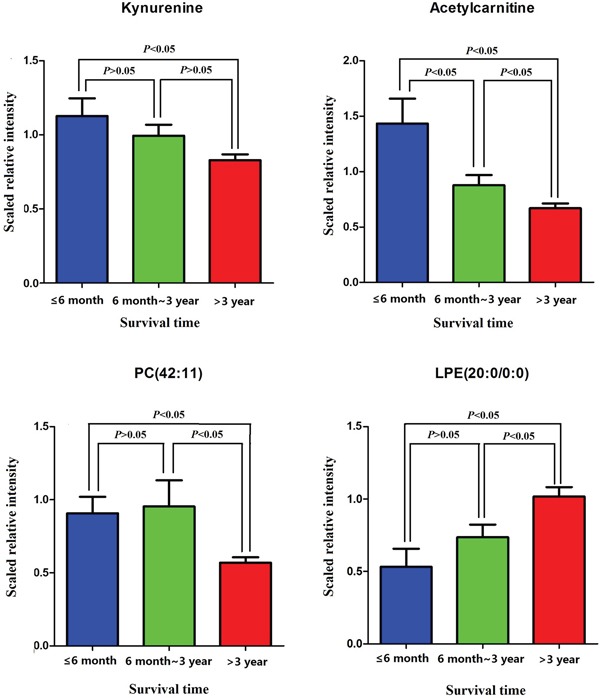
Histogram displaying the temproal patterns of each predictive metabolite among three different survival times

**Figure 6:**
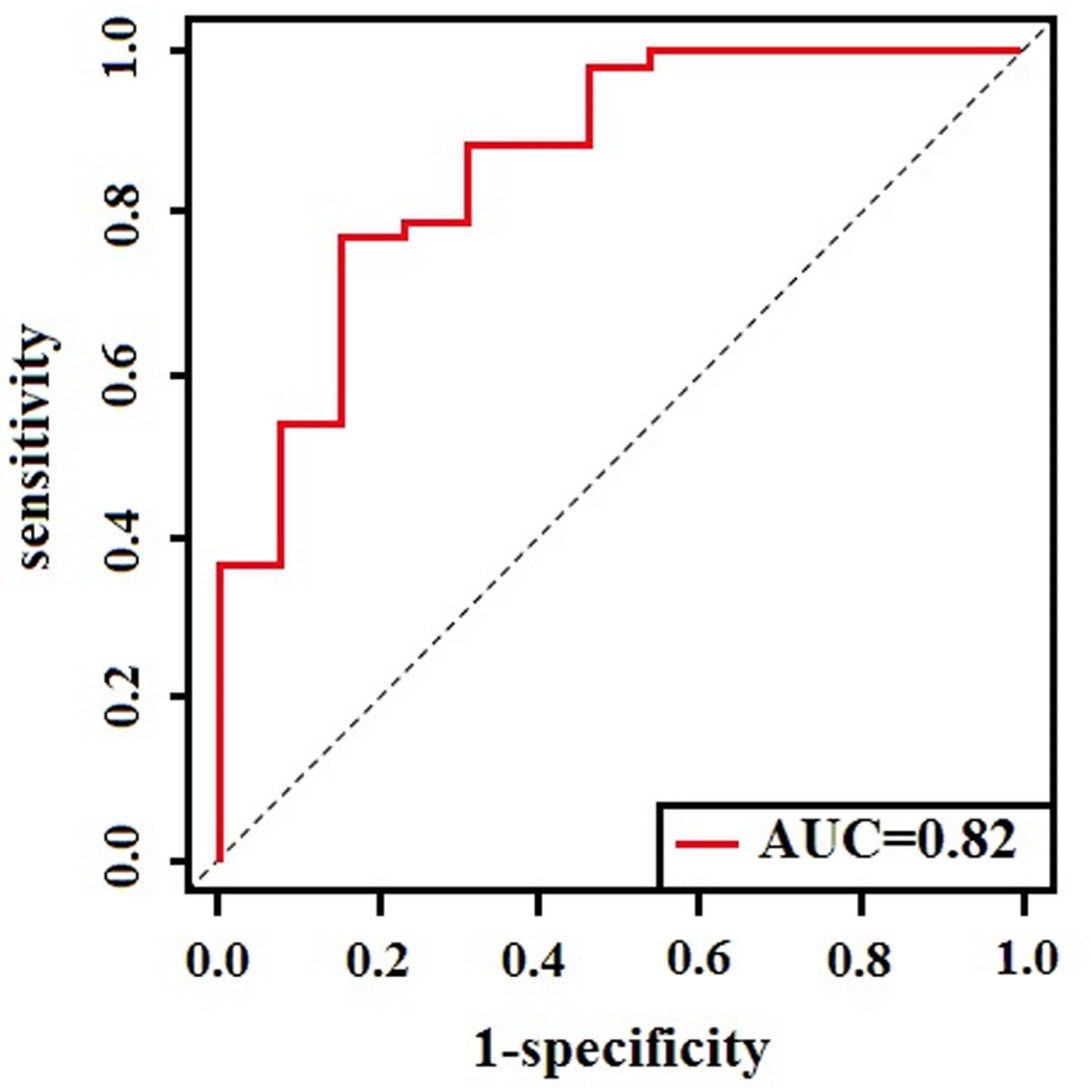
Risk scoreROC curve to evaluate the predictive accuracy between short-term mortality and long-term survival

Further investigation of the temporal patterns of four predictive biomarkers and their discriminating ability in EOC progression, i.e. the short-term mortality and long-term survival, has shown that the four potential predictive biomarkers were significantly altered in short-term mortality compared to long-term survival patients (P<0.05). Ninety-eight EOC patients were divided into three groups by survival time: short-term mortality (n=13), medium survival (n=33), and long-term survival (n=52). Histograms presented in Figure 5 indicated that Acetylcarnitine had significant alterations in any two groups, while PC (42:11) and LPE(22:0/0:0) were significantly altered in short-term mortality and medium survival. Patients with long-term survival showed increased plasma relative intensity of LPE (20:0/0:0) and decreased relative intensity of Kynurenine, Acetylcarnitine, and PC(42:11). In addition, AUC value was calculated to evaluate the predictive performance of short-term mortality and long-term survival by these biomarkers. The combination of the four metabolites could discriminate the short-term mortality and long-term survival in EOC patients with the AUC value of 0.82 (Figure 6), and the clinical characteristics were listed in Supplementary Table 3.

## DISCUSSION

Metabolites could be regarded as the ultimate response to the process of diseases occurring in living organisms. Recent studies revealed that dysregulated metabolism was associated with the prognosis of human cancers and utilized metabolites, as markers to predict survival. To our knowledge, this is the first study specifically designed to evaluate the predictive performance of survival for EOC patients, based on the pretreatment plasma metabolomics. It would be considerably meaningful to develop a metabolite-based prognostic model to predict the OS for ovarian cancer. A study has been reported that the peak mortality occurred approximately between 2 years and 3.5 years after diagnosis for EOC patients, who carried BRCA1 or BRCA2 mutations and noncarriers, respectively [27]. In ovarian cancer patients, 3 years were chosen as the peak mortality. In the present study, a non-targeted UPLC/MS plasma metabolism method was used to explore the metabolic characteristics related to the survival of EOC patients and to screen for meaningful and vital survival predictors for the projection of 3-year mortality. We identified 28 metabolites in ESI+ mode and 12 metabolites in ESI- mode related to the OS for EOC. Kynurenine, Acetylcarnitine, PC (42:11), and LPE (20:0/0:0) were selected as the vital survival potential predictive biomarkers. In addition, we constructed a risk score: four potential biomarkers together with pathological stage, and also evaluated the predictive accuracy of 3-year survival with the time-dependent AUC value of 0.80. Meanwhile, we described the temporal patterns of those four predictive metabolites in the disease progress and evaluated the discrimination ability of short-term mortality and long-term survival with the AUC of 0.82. It is meaningful to distinguish the short-term mortality from the long-term survival, because patients with short-term mortality are in need of intensive care and are facing significant economic costs.

Currently, several researches have utilized the clinical information to predict the prognosis of ovarian cancer. However, due to the heterogeneity of ovarian cancer, it is difficult to predict the clinical outcomes of patients with simple demographic and clinical characteristics. In terms of CA125, Gupta et al. had reviewed the role of CA125 in predicting ovarian cancer survival and concluded that the results from different studies were sometimes contradictory, and the cut off values were always different [28]. A study demonstrated that patients with preoperative values below 65 U/ml had significantly longer survival compared to those above 65 U/ml in stage I ovarian cancer [29]. However, Osman et al. concluded that CA125 level did not correlate with survival [30], which is consistent with our study. Whether the preoperative CA125 has the utility as a prognostic indicator of survival, as well as of diagnosis, still needs further study. In addition, primary cytoreductive surgery, followed by chemotherapy, is the international standard of care for women with advanced ovarian cancer. However, after controlling the important metabolic prognostic factors, there was no significant correlation between chemotherapy and OS in our study. It is likely that our predictive biomarkers were not associated with chemotherapy sensitivity, which still requires further research.

Our team previously reported that L-tryptophan metabolism, including L-tryptophan, 3-indolepropionic acid, kynurenine, and 5-hydroxyindoleacetaldehyde, was disturbed in EOC patients [31]. The other study from our research group demonstrated that lower L-tryptophan and higher kynurenine were associated with recurrence of EOC [32]. We observed a similar result for association between metabolites and cancer mortality in this study, i.e. poor OS associated with the increased plasma kynurenine. It has been reported that tryptophan is catabolized through Kynurenine Pathway (KP) to vital energy cofactor, nicotinamide adenine dinucleotide (HAD^+^), which could promote cellular growth [33]. Kynurenine is the first catabolite produced from tryptophan by IDO1/2 and TDO2, which is biologically active in various physiological mechanisms, and it has been identified as an endogenous ligand for the aryl hydrocarbon receptor (AhR) involved in diverse cellular functions such as cellular differentiation and proliferation [34]. Moreover, evidence has emerged to suggest that upregulated IDO1 and TDO2 expression within the tumor was correlated with decreased OS of patients with glioma [35, 36]. IDO has been known for its role in tumor-induced immune escape [37]. Several studies have previously shown that kynurenine/tryptophan index is a useful prognostic indicator for patients with human cancers, such as lung cancer [38], breast cancer [39], and acute myeloid leukemia [40]. de Jong et al. has revealed that kynurenine and kynurenine/tryptophan ratios were higher in the pretreatment serum samples from patients with endometrial, ovarian, and vulvar cancers, than that of controls [41]. Therefore, kynurenine would be a predictive biomarker in predicting the OS in pretreatment ovarian cancer.

Acetylcarnitine is an amino acid derived from the body itself and certain food. It is synthesized in the brain, liver, and kidney by the enzyme Acetylcarnitine transferase. Acetylcarnitine could influence the cholinergic system as a cholinergic receptor agonist, promoting synthesis and release of acetylcholine [42], and it has been reported that Acetylcarnitine dysregulation usually occurs in neurological disorders [43–45], Zhao et al. have demonstrated that low Acetylcholinesterase expression was discovered in hepatocellular carcinoma tissue and had significantly associated with high risk of cancer recurrence and poor survival [46]. As we know, Acetylcholinesterase was used to decompose acetylcholine, and low Acetylcholinesterase may lead to the accumulation of acetylcholine in the hepatocellular carcinoma, and our current study suggested that high intensity of Acetylcarnitine was associated with poor survival, which indicated that high Acetylcarnitine might promote the release of acetylcholine in ovarian cancer. More generally, Acetylcarnitine participates in the oxidation of fatty acids by the means of facilitating the uptakes of acetyl-CoA into the mitochondria and produces energy to maintain the process of cancer.

Recent accumulating evidence suggests that lipid metabolism is disturbed in a variety of cancers [47, 48], because lipids play important roles in constructing cell membranes, cell signaling, and producing energy to sustain the tumor cell proliferation. Zhang et al. has revealed plasma lipidomic alterations in ovarian cancer in Asian women [48], while Park et al. has validated that LPE, a metabolite of phosphatidylethanolamine (PE), not only increases intracellular Ca2+ via LPA1 and CD97 but also correlates with cell proliferation and migration in breast cancer cells [49]. In the present study, we provided evidence that there exists a relationship between lipids and OS in ovarian cancer, especially PC, LPC, LPE, and ceramides, and we chose the PC(42:11) and LPE(22:0/0:0) as the important potential biomarkers to predict the OS of ovarian cancer. We concluded that poor survival was due to the increase of PC(42:11) and with the decrease of LPE(22:0/0:0).

To our knowledge, this is the first study to prospectively assessed the mortality risks of ovarian cancer patients after diagnosis, based on the pretreatment plasma metabolomics. However, our study had several limitations. We have only considered cycles of chemotherapy as the predictive factor for treatment, i.e. initial treatment of cytoreductive resection, which turns out to be one of the limitations. Another limitation is lack of clinical information for some patients, which would influence the stability of the predictive model to some extent.

In summary, we demonstrate that the intensities of the plasma metabolites are closely related with the OS in EOC patients and identify a panel of four metabolites as potential biomarkers to predict the three-year survival of EOC patients. Further analysis reveal that metabolite-based predictive risk scores are independent of clinical predictors for EOC survival. Finally, the temporal patterns of each potential predictive biomarker and their predictive performance were observed. Our study would provide information for further study about EOC survival mechanism, and the accurate prognosis prediction could guide the appropriate treatment clinically.

## MATERIALS AND METHODS

### Study population

This study was approved by the Ethics Committee of the Tumor Hospital of Harbin Medical University, and all patients signed informed consents before the study began. Participants who were suffering from metabolic diseases, liver diseases, kidney diseases, or any other cancers were excluded. We enrolled 120 EOC patients who were diagnosed and received surgery between August, 2009 and April, 2013 and obtained the follow-up information from the hospital follow-up center during April 1st-5th, 2016. Patients lost to follow-up or without definite endpoint information, e.g. unclear death time, were excluded from this study. In total, nighty-eight EOC patients with survival and death information were enrolled in this study. For patients who received surgery and died in three years, we are still gathering the information of the survival time after surgery/treatment and the cause of death.

### Sample collection

Plasma samples were collected from pretreatment primary ovarian cancer patients at the Department of Gynecology of Harbin Medical University Tumor Hospital (Harbin, China). Fasting venous blood samples were collected using vacuum blood collection tube that contained anticoagulant dipotassium EDTA. Plasma was separated by centrifugation at 1,323g for 10min and the supernatant was stored at -80°C until further analysis.

### Sample preparation

To ensure the stability and repeatability of ultra-performance liquid chromatography mass spectrometry (UPLC/MS) systems, a total of 10 blank samples and 10 quality control (QC) samples were used in this study. All the plasma samples were thawed at 4°C and a volume of 200 μl of plasma was mixed with 600 μl of acetonitrile at 4°C. Mixed samples were centrifuged at 1,323g for 10 min. The supernatant was transferred into a clear vial and dried in a vacuum rotary dryer. The residue was dissolved in 100 μl acetonitrile/water (1:3, v/v), vortex-mixed for 5 min, then centrifuged at 1,323g for 15 min, and the supernatant was held for further analysis.

### Chromatography

A 10 μl aliquot of the pre-treated sample was injected into a 2.1 × 100 mm ACQUITY UPLC BEH C18 column using a UPLC system. This UPLC system was performed using acetonitrile containing 0.1% formic acid (solvent A) and water containing 0.1% formic acid (solvent B) as the mobile phase at a flow rate of 0.3 ml/min at 40°C. A linear mobile phase gradient was used as follows: 1% A, held for 0.5 min; 0.5-4.0 min, increased to 15% A; 4.0-4.5 min, increased to 55% A; 4.5-11.5 min, increased to 90% A; 11.5-12.0 min, increased to 99% A; and 12.0-15.0 min, held at 99% A. After the analytical run, the mobile phase was returned to 1% A in 0.1 min and equilibrated at 1% A for 1 min.

### Mass spectrometry

Mass spectrometry was performed with an Agilent 6520-QTOF, equipped with electrospray ionization (ESI) source operating at positive-ion (ESI+) and negative-ion (ESI-) electrospray ionization mode. The capillary voltage was 4.0 kV at ESI+ mode and 3.5 kV at ESI- mode. Nitrogen was used as the dry gas, and the desolvation gas flow was set at 10 l/min. The desolvation temperature was set at 330°C. Centroid data were collected in the full scan mode from 50 to 1000 m/z.

### Data preprocessing and annotation

Raw data were converted into mzdata-format files by MassHunter Qualitative Analysis Software and then imported to the XCMS packages in R for preprocessing. The parameters were the same as previous studies [31]. CAMERA in R was used for annotation of the preprocessing results. Isotopic peaks were excluded prior to statistical analysis.

### Metabolites related to survival

The primary outcome was assessed using three-year OS, which was defined as time from diagnosis to death due to any causes or last follow-up of patients that were still alive [50]. The preliminary association between the metabolites and survival time was assessed by univariate Cox proportional hazards regression, and the corresponding lfdr value was estimated in order to correct the multiple comparisons. Metabolites with lfdr < 0.05 were considered to be significantly associated with survival. RSF: a multivariate survival analysis method was performed to calculate the relative variable importance (vimp) value for the significance of metabolites in survival analysis, which can effectively deal with the co-linearity and the interaction among variables. We ranked the vimp values in descending order, and top ten metabolites were chosen for further study. For the purpose of prediction, the predictive metabolites were as far as possible to provide complementary information between each other, and we selected four metabolites as the potential predictive biomarkers, based on the spearman correlation coefficient (CC) lower than 0.5 between each pair of metabolites. The relative intensity of these four metabolites was categorized into low, medium, and high relative intensity groups, based on their corresponding 25^th^ and 75^th^ percentiles as cutoffs, respectively. Survival curves were calculated by the Kaplan-Meier method and compared using the log-rank test for the difference among low, medium, and high relative intensity groups for each metabolite.

### Risk score and its predictive performance

In order to facilitate the application in clinical practice, a risk score was defined as a linear combination of four metabolites together with clinical information. The scaled intensity of those four metabolites alone was calculated, and coefficient for each metabolite in the risk score was weighted by their respective Cox regression coefficients, based on this scaled relative intensity. Time-dependent area under the receiver operating characteristic (ROC) curve, allowing characterization of diagnostic accuracy for censored survival outcomes, was explored to evaluate the predictive accuracy [51].

### Discrimination performance of potential biomarkers between short-term mortality and long-term survival groups

Ninety-eight EOC patients were divided into three groups by survival time: less than 6 months, between 6 months and 3 years, and longer than 3 years. Each category was defined as short-term mortality, medium survival, and long-term survival, separately. After that, in order to explore if four metabolites were different across the groups, we utilized Student's t test to compare the alteration of the four metabolites between each two groups. In addition, further study was performed to explore whether those four metabolites had the potential discrimination ability between short-term mortality and long-term survival. We performed 5-fold cross validation to assess the predictive accuracy of the combination of four predictive biomarkers between short-term mortality and long-term survival fitting with Random Forest model. The statistical analysis was performed in the statistical program R (http://www.r-project.org).

## SUPPLEMENTARY MATERIALS FIGURES AND TABLES





## Abbreviations

EOC: epithelial ovarian cancer
UPLC/MS: ultra-performance liquid chromatography mass spectrometry
QC: quality control
ESI: electrospray ionization
lfdr: local false discovery rate
RSF: Random Survival Forest
vimp: the relative variable importance
CC: correlation coefficient
ROC: receiver operating characteristic
m/z: Measured mass to charge ratio
RT: Retention time
HR: Hazard ratio
CI: Confidence interval
PC: Phosphatidylcholine
LPE: lysophosphatidylethanolamine
CA125: cancer antigen 125
KP: Kynurenine Pathway
HAD^+^: nicotinamide adenine dinucleotide
AhR: aryl hydrocarbon receptor
PE: phosphatidylethanolamine.
