# Supplementary material for: Metabolic profiling and novel plasma biomarkers for predicting survival in epithelial ovarian cancer
Source: Oncotarget. 2017 Mar 31;8(19):32134–46. doi: 10.18632/oncotarget.16739 (PMC5458273; doi:10.18632/oncotarget.16739)
Supplement: Supplementary file 1 [file oncotarget-08-32134-s001.pdf]

Metabolic profiling and novel plasma biomarkers for predicting survival in epithelial ovarian cancer

SUPPLEMENTARY FIGURE AND TABLES

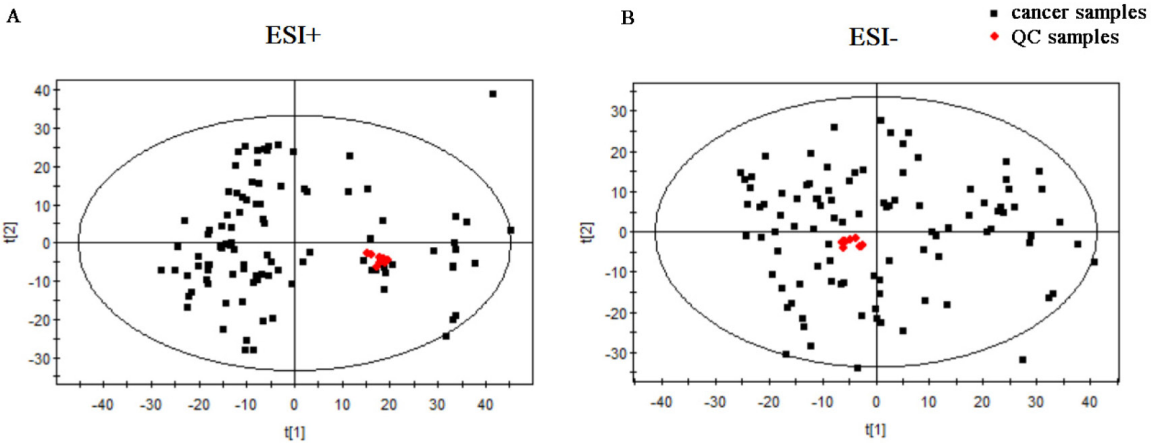

Supplementary Figure 1: (A) PCA score for cancer samples and QC samples in ESI+ mode. **(B)** PCA score for cancer samples and QC samples in ESI- mode.

Supplementary Table 1: Demographic and clinical characteristics of EOC patients in this study

Characteristics	N=98
Age (year)	
<50	34(34.69)
≥50	64(65.31)
Menopause(pre/post)	
Pre	40(40.82)
Post	58(58.16)
Undocumented	1(1.02)
CA125(median,range) (U/ml)	543.30(9.08-5000)
≤500	47(47.96)
>500	51(52.04)
Chemotherapy (cycle)	6(0-20)
<6	23(23.47)
≥6	40(40.82)
Undocumented	35(35.71)
FIGO stage	
I	9(9.18)
II	9(9.18)
III	56(57.14)
IV	7(7.14)
Undocumented	17(17.35)
Histology type	
Serous	46(46.94)
Others	10(10.20)
Undocumented	42(42.86)
Histology differentiation	
Well	4(4.08)
Moderately	11(11.22)
Poorly	50(51.02)
Undocumented	33(33.67)

Supplementary Table 2: Metabolites related to EOC survival based on univariate Cox regression analysis

See Supplementary File 1

Supplementary Table 3: Demographic and clinical characteristics of EOC patients of short-term mortality and long-term survival

	≤6 month(n=13)	6month~3year(n=33)	>3year(n=52)
Age(median,range)			
<50	3(23.08)	12(36.36)	19(36.54)
≥50	10(76.92)	21(63.64)	33(63.46)
Menopause			
Pre	9(69.23)	20(60.61)	28(53.85)
Post	4(30.77)	12(36.36)	24(46.15)
Undocumented	0(0.00)	1(3.03)	0(0.00)
CA125(median,range)			
≤500	7(53.85)	18(54.55)	22(42.31)
>500	6(46.15)	15(45.45)	30(57.69)
Chemotherapy			
<6	3(23.08)	8(24.24)	12(23.08)
≥6	0(0.00)	7(21.21)	33(63.46)
Undocumented	10(76.92)	18(54.55)	7(13.46)
FIGO stage			
I	0(0.00)	1(3.03)	8(15.38)
II	0(0.00)	2(6.06)	7(13.46)
III	8(61.54)	20(60.61)	28(53.85)
IV	2(15.38)	4(12.12)	1(1.92)
Undocumented	3(23.08)	6(18.18)	8(15.38)
