# Supplementary material for: Metabolic profiling and novel plasma biomarkers for predicting survival in epithelial ovarian cancer
Source: Oncotarget. 2017 Mar 31;8(19):32134–46. doi: 10.18632/oncotarget.16739 (PMC5458273; doi:10.18632/oncotarget.16739)
Supplement: Supplementary file 2 [file oncotarget-08-32134-s002.doc]

**Table S2. Metabolites related to EOC survival based on univariate Cox regression analysis**

| num | m/z | RT(min) | Name | Vimp | ppm | *P* value | lfdr |
| --- | --- | --- | --- | --- | --- | --- | --- |
| Positive-ion electrospray ionization mode (ESI+) | | | | | | | |
| 1 | 204.1221 | 1.88 | Acetylcarnitine | 0.010745 | 4.69 | <0.00001 | 0.00084 |
| 2 | 209.0916 | 3.66 | Kynurenine | 0.012115 | 2.35 | 0.00019 | 0.00670 |
| 3 | 290.1335 | 9.09 | Ophthalmic acid | 0.000583 | 4.09 | 0.00037 | 0.00940 |
| 4 | 452.2775 | 8.49 | LPE(16:1/0:0) | -0.00058 | 0.69 | 0.00252 | 0.03174 |
| 5 | 468.3085 | 8.14 | LPC(14:0) | 0.003928 | 0.02 | 0.00027 | 0.00670 |
| 6 | 476.2774 | 8.18 | LPE(18:3/0:0) | 0.003658 | 0.44 | 0.00132 | 0.02707 |
| 7 | 480.343 | 10.38 | LPC(P-16:0) | -2.32E-05 | 3.90 | 0.00270 | 0.03174 |
| 8 | 482.3241 | 8.93 | LPC(15:0) | 0.002986 | 0.08 | 0.00008 | 0.00527 |
| 9 | 494.3243 | 8.52 | LPC(16:1) | 0.000451 | 0.32 | 0.00017 | 0.00670 |
| 10 | 496.3407 | 9.86 | LPC(16:0) | -0.00018 | 1.83 | 0.00033 | 0.00683 |
| 11 | 506.3597 | 11.94 | LPC(P-18:1) | 0.000898 | 1.63 | 0.00018 | 0.00670 |
| 12 | 508.3389 | 9.38 | LPE(0:0/20:1) | 0.000551 | 1.75 | 0.00033 | 0.00670 |
| 13 | 508.3762 | 12.40 | LPC(P-18:0) | 0.002882 | 0.05 | 0.00099 | 0.02014 |
| 14 | 510.3557 | 10.88 | LPC(17:0) | 0.001442 | 0.51 | 0.00141 | 0.02707 |
| 15 | 518.3241 | 8.22 | LPC(18:3) | 0.001911 | 0.08 | 0.00114 | 0.02707 |
| 16 | 520.3375 | 9.75 | LPC(18:2) | 0.001769 | 4.40 | 0.00119 | 0.02707 |
| 17 | 522.3558 | 9.96 | LPC(18:1) | 0.000419 | 0.69 | 0.00197 | 0.03174 |
| 18 | 524.3721 | 11.94 | LPC(18:0) | 0.000142 | 1.93 | 0.00031 | 0.00670 |
| 19 | 530.3238 | 10.42 | LPE(22:4/0:0) | 0.000911 | 0.64 | 0.00261 | 0.03174 |
| 20 | 538.3865 | 12.81 | LPE(22:0/0:0) | 0.00705 | 0.45 | 0.00008 | 0.00527 |
| 21 | 546.3556 | 9.72 | LPC(20:3) | 0.001403 | 0.29 | 0.00121 | 0.02707 |
| 22 | 552.4026 | 13.55 | LPC(20:0) | 0.005204 | 0.38 | 0.00158 | 0.02707 |
| 23 | 562.4222 | 12.95 | CerP(d18:1/12:0) | 0.002752 | 1.64 | 0.00034 | 0.00940 |
| 24 | 570.3547 | 9.96 | LPC(22:5) | 0.000711 | 1.30 | 0.00286 | 0.03174 |
| 25 | 572.3706 | 10.48 | LPC(22:4) | 0.000537 | 0.86 | 0.00173 | 0.02707 |
| 26 | 752.5227 | 14.01 | PC(14:0/20:5) | 0.001561 | 0.26 | 0.00051 | 0.01159 |
| 27 | 804.5535 | 14.01 | PC(18:3/20:4) | 0.004077 | 0.38 | 0.00139 | 0.02707 |
| 28 | 852.553 | 13.33 | PC(22:6/20:5) | 0.00991 | 0.94 | 0.00249 | 0.03174 |
| Negative-ion electrospray ionization mode (ESI-) | | | | | | | |
| 29 | 104.0357 | 1.66 | L-Serine | -0.00044 | 3.91 | 0.00295 | 0.0348 |
| 30 | 103.0401 | 3.09 | 3-Hydroxybutyric acid | 0.00048 | 0.54 | 0.00001 | 0.00616 |
| 31 | 117.0557 | 4.48 | 3-Hydroxy-2-methyl-[R-(R,R)]-butanoic acid | 0.001333 | 0.05 | 0.00009 | 0.00842 |
| 32 | 436.2836 | 10.28 | PE(P-16:0e/0:0) | 0.002582 | 0.63 | 0.00017 | 0.00842 |
| 33 | 474.2628 | 8.13 | LPE(18:3/0:0) | 0.001489 | 0.44 | 0.00229 | 0.02857 |
| 34 | 502.2947 | 9.64 | LPE(20:3/0:0) | -0.00149 | 1.61 | 0.00414 | 0.04491 |
| 35 | 504.3098 | 9.05 | LPE(20:2/0:0) | 0.000569 | 0.52 | 0.00027 | 0.00842 |
| 36 | 506.3253 | 10.28 | LPE(20:1/0:0) | -0.00091 | 0.22 | 0.00456 | 0.04491 |
| 37 | 508.3413 | 11.90 | LPE(20:0/0:0) | 0.000257 | 0.91 | 0.00123 | 0.02545 |
| 38 | 564.5327 | 9.05 | Cer(d18:0/18:1) | -6.68E-05 | 6.01 | 0.000272 | 0.00842 |
| 39 | 566.5491 | 10.28 | Cer(d18:0/18:0) | -0.00139 | 4.67 | 0.002903 | 0.0348 |
| 40 | 1091.728 | 11.90 | Ganglioside GA2 (d18:1/18:0) | -0.001 | 6.31 | 0.00246 | 0.02857 |

Abbreviations: Measured mass to charge ratio (m/z); Retention time (min, RT); Relative variable importance (Vimp); Local false discovery rate (lfdr).
